# Investigation and genetic confirmation of the *Cryptosporidium species* in fish handlers in Baghdad city

**DOI:** 10.5455/javar.2025.l866

**Published:** 2025-03-22

**Authors:** Noor Majed Obead, Amer Rasool Alhaboubi

**Affiliations:** Department of Parasitology, College of Veterinary Medicine, University of Baghdad, Baghdad, Iraq

**Keywords:** *Cryptosporidium*, fish handlers, Iraq, SSU, nested PCR

## Abstract

**Objective:**

The present study aims to investigate molecular confirmation for *Cryptosporidium* species in fish handlers in Baghdad City, central Iraq.

**Materials and Methods:**

Sixty stool samples were collected between early November 2023 and late April 2024. All samples were examined phenotypically using a modified Ziehl-Neelsen stain and genotypically (nested polymerase chain reaction technique) based on a partial sequence of 18S rRNA genes with sequencing and phylogenetic tree analysis.

**Results:**

The total molecular results identified *Cryptosporidium parvum* with an infection rate of 45% (27/60). A higher infection rate of 51.9% (14/27) was found in the age group between 15 and 35 years, and male handlers recorded a lower infection rate (45%) than females (41.6%). April had a higher elevation in the infection rate of 60% (6/10) than other months.

**Conclusion:**

The *C*.* parvum* was the only species found in fish handlers, and these local isolates have higher similarity with other isolates of China and Iran.

## Introduction

*Cryptosporidium* is a food-borne protozoan parasite that infects a wide range of hosts, including wild and domestic animals and humans, causing millions of illnesses every year [1]. The genus *Cryptosporidium* was identified with 44 species known to infect amphibians, reptiles, birds, and fish. In addition, more than 70 genotypes have been described in different hosts, and the human disease of Cryptosporidiosis is frequently the cause by far. The most common species recognized globally are *Cryptosporidium parvum* and *Cryptosporidium hominis*. They were described in fish and confirmed the presence of *Cryptosporidium nasoris* in the intestines of tropical aquatic life [2]. It had been found that these protozoa were in wild and cultured freshwater and fish worldwide, and other studies detected the infection of *Cryptosporidium* in several fish species, such as carp (14.3%), black Nile catfish (10.0%), North African catfish (20.0%), Nile tilapia (30.0%), and Liza abu (12.2%) [3,4]. It is frequently found in rivers, recreational and sewage water, and the fecal-oral transmission mode for *Cryptosporidium* makes excellent use of water as a medium for its spread [5].

The main clinical signs caused by the parasite are severe watery diarrhea, which mostly occurs in immunocompromised patients and toddlers under five years old. The prevalence of infection includes a wide range of vertebrates, such as marine life (fish and amphibians), birds, and mammals. It can be contiguous with other parasitic and bacterial diarrheal infections [6–8].

Although many Iraqi studies focused on the presence of *Cryptosporidium* in human, cattle, equine, and bird hosts, very limited studies were conducted in Iraq that have reported the detection of this parasite in fish [9–12]. Because of the lack of molecular data about this protozoan parasite prevalence and identification in fish hosts in Iraq, the present work was conducted to cover the prevalence of *Cryptosporidium* spp. in fish handlers in Iraq. Herein, the study aimed to investigate the *Cryptosporidium* infection in fish handlers (male and female) in the city of Baghdad center and suburban area by conventional and nested polymerase chain reaction (PCR) tests.

## Materials and Methods

### Ethical approval

This study was approved according to research guidelines and laboratory rules and regulations of the College of Veterinary Medicine, University of Baghdad (P.G. 1296 on July 9th, 2024).

### Sample collection

Sixty stool samples were randomly collected from male and female fish handlers of different ages at Baghdad markets. Samples include people working on fish cleaning and preparation. The sample providers signed a questionnaire and agreement form, and the study covered six months from early November 2023 to the end of April 2024. Stool samples were preserved in a cooler, sealed in one-use, sterile plastic labeled containers, and transmitted to the Department of Parasitology Lab, College of the Veterinary Medicine/University of Baghdad. The sample was divided into two portions. The first one applied to the traditional parasitic examination by using the flotation concentration method employing Sheather’s sugar solution technique and modified Ziehl-Neelsen stain for positive samples and microscopic examination by oil immersion lens for the detection of the parasite oocyst, while the second part was for molecular analysis.

### DNA isolations

The Presto™ Stool DNA Extraction Kit was used for stool DNA extraction from 20 human stool samples, and it was quantified by Nanodrop Spectrophotometer (Thermo, USA) with a concentration range between 5 and 130 ng/μl.

### Amplifications and primers

An amplification primer applied herein included a nested reaction PCR primer for the detection of *Cryptosporidium* spp. Small subunit ribosomal RNA gene. These primers were provided by the Scientific Researcher Co. Ltd, Iraq. A pair was used as PCR primers. The Forward (small subunit [SSU]-F1) 5'-TTC TAG AGC TAA TAC ATG CG-3' and Reverse SSU-R1 5'-CCC TAA TCC TTC GAA ACA GGA-3' were utilized in the first round of PCR, capturing a segment of 1,325 bp within the gene fragment. The second primers, SSU-F2 (5'-GGA AGG GTT GTA TTT ATT AGA TAA AG-3') and SSU-R2 (5'-AAG GAG TAA GGA ACA ACC TCC A-3'), were used in the second run, targeting a fragment estimated to be 815 bp of ribosomal DNA [13].

### PCR conditions

The PCR kit GoTaq^®^ PCR Green Master Mix (Promega, USA) was applied for both reactions. For the primary reaction, a total volume of 25 μl was used. A 12.5 μl of the 2x master mix was added with 5 μl of the template. 2 μl of each upstream and downstream primer with a concentration of 10 pmol and 3.5 μl of Nuclease-Free water were added to the total reaction volume.

Thermocycler conditions begin with denaturation (95°C) for 5 min, then followed by 35 cycles of denaturation (95°C) for 30 sec. DNA annealing (55°C) for 30 sec, extension at 72°C for 2 min, and finally extension (72°C) for 5 min was followed by a hold-on phase at 4°C [13].

In the nested reaction round, 12.5 μl of the 2x master mix is added to 1 μl of primary PCR product. Then, one μl each of 18SrRNA gene forward and reverse primers 10 pmol (inner set) and 9.5 μl from the water of PCR complement the reaction volume. The nested amplification protocol, except for the annealing temperature, was used as above at 57°C. All amplicons were electrophoresed through a 1.5% agarose gel alongside a 100 bp marker (iNtRON, Korea), stained with ethidium bromide, and visualized under an ultraviolet transilluminator.

### DNA sequencing and phylogenetic analysis

The resulting PCR products (five samples) were sequenced at Macrogen Inc. in South Korea. The DNA sequencing data analysis was performed using the National Center for Biotechnology Information (NCBI) web. Nucleotide-BLAST^®^ program to calculate significance and compare the similarities between sequence databases. The constructed phylogenetic tree was carried out by using the software Molecular Evolutionary Genetics Analysis (Mega X version) [14]. Multiple sequence DNA fragment alignment using the ClustalW alignment tool, and the genetic evolutionary distances were estimated by the UPGMA tree construct on the Max Composite Likelihood method. The resulting *Cryptosporidium* spp. isolates were submitted to the NCBI-GenBank to assign accession numbers. The tree includes all sequences with a polar-clad gram using the genetic tree.

### Statistical analysis

The study used “SPSS Version 23.0” for Windows to validate the connection between this parasite and each of the researcher’s factors using the Chi-square test. Microsoft Excel 365 is constructed differently to be significant at levels *p* ≤ 0.05 and *p* ≤ 0.01.

## Results and Discussion

The results emphasize some molecular and traditional investigations underlying the local and regional *Cryptosporidium* isolates.

**Figure 1.**
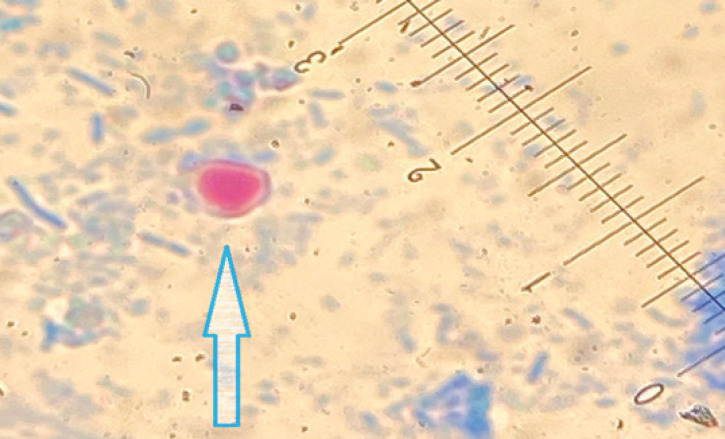
The figure shows *Cryptosporidium* oocyst (cyan arrow) stained by Modified Ziehl-Neelsen stain (100×).

The results expose a clear prevalence of inducing infection in fish handlers in Baghdad city. Microscopically, the parasitic stage (oocysts) morphology appears as oval (4.6–5.5 × 3.8–4.7 μm) by traditional techniques (modified Ziehl-Neelsen) and is surrounded by a red circular hallow shape and blue backdrop (Fig. 1).

Among sixty stool samples (male and female) examined, the infection rate of *Cryptosporidium* was 45% (27/60). Male handlers recorded a higher infection rate of 45.8% (22/48) than females at 41.6% (5/12), with significant differences appearing in Table 1.

The result differed from the infection rate in fish men recorded in Tikrit city, which was 6.6%. These results differed from earlier study findings, indicating fishermen in Tikrit City were infected with a total infection rate of 6.6%.

*Cryptosporidium* spp., recorded in aquatic habitats and spread to humans through fish consumption, has recently been highlighted as a potential fish-borne disease. The fish harbor host-specific species/genotypes and species like zoonotic *C*.* parvum* and *C. hominis* [15,16]. These divergences may be recognized due to regional differences, general hygiene social practices, environmental changes, or other factors that vary.

The incidence of patients is more severe than in animals, and the rates of infection are different in America (15%) and may reach 100% in third-world countries; it also spreads in overcrowded areas such as children’s kindergartens and play areas [17]. The results were consistent with a similar study in Baghdad handlers of domestic pigeons with an infection rate of 55.5% [18], and in Mosul City, they observed an infection rate of 43.56% (34/78) [19].

### Infection rates of Cryptosporidium according to age

The *Cryptosporidium* infection rates shown in four groups of different ages among handlers of fish reveal different patterns. The higher rate was found in humans between the age group of 15 and 35 years, mainly fish sellers and dealers (51.85% [14/27]). In the third group (35–55 years), the infection rate was quite reduced, with 44.4% (8/18). Among individuals younger than 15 years (family of the handler), a relatively low but still notable infection rate of 33.3% (2/6) was recorded. As well as the last age group (55 years old and above) exhibited a rate of infection at 33.3% (3/9) out of the total examined handlers with a significant difference (*p *≤ 0.01). These findings explain a certain relation between age and the revelation of parasite infection as seen in Table 2.

These results agree with other survey studies on birds conducted in Diyala and Babylon provinces [20,21]. In contrast, in earlier studies, people of young ages were more susceptible to the diseases than older ones [22]. Some studies in the Arab world have indicated that infection rates in children under five are 4.8% in Saudi Arabia [23]. Tikrit’s and Ramadi’s rates were 4.2% and 39.13%, respectively [24]. In Mosul provinces, the infection was 18.8% of children [25]. Alaa and Ghaidaa (2011) [26] reported infection in Diwania city by 17.5% within the diarrhetic children group and 8.3% in other clear symptom samples. Emphasizes the effect of considering age-related factors when evaluating *Cryptosporidium* infection. However, here we were trying to link the recorded infection rates of handlers (animals, poultry, and even fish) of distinct age groups, which may exhibit different susceptibilities to illness.

**Table 1.** Infection rates of *Cryptosporidium* spp. in fish-handlers according to gemders.

Gender	No. of examined	No. of infected	(%)
Males	48	22	45.8
Females	12	5	41.6
Total	60	27	45
*p*-value 0.0001*			

* Significant differences.

### Infection rate of Cryptosporidium spp. according to months

During the study period, infection rates among fish handlers constantly appeared approximately altered. Prevalence was regularly stated during the six months. However, the prevalence stated distinctive patterns, with significant differences (*p* ≤ 0.01) between infection rates, as in Table 3. During April, the rates elevated to 60% (6/10), indicating abundant prevalence during these months. In February and March, the infection rate remained notably high, at 50% (4/8) and (6/12). The months of December and November show considerably lower infection rates of 30% (3/9) and 40% (4/10), respectively, with significant differences (*p *< 0.01). These variations in infection rates determine a clear seasonal influence on parasites dominated among fish handlers.

The effect factor of months of the year was reported previously in the parasite prevalence. Locally, Sayal [26] partially agrees with the current outcome, a recorded rate of 24.1% in humans at Al Najaf city during February. These values support the premise that the colder months (winter) of January and February are usually accompanied by elevated *Cryptosporidium* transmission [27]. Al-Jawasim and Al-AKhaled [27] reported a 30% infection rate among humans in September by recording a different seasonal onset in a local study. The results mostly point out the variability of parasite occurrence and clarify the importance of environments and general health conditions, along with human behaviors (social and traditional events) concerning seasonal incidents.

### Molecular analysis of fish handlers

**Figure 2.**
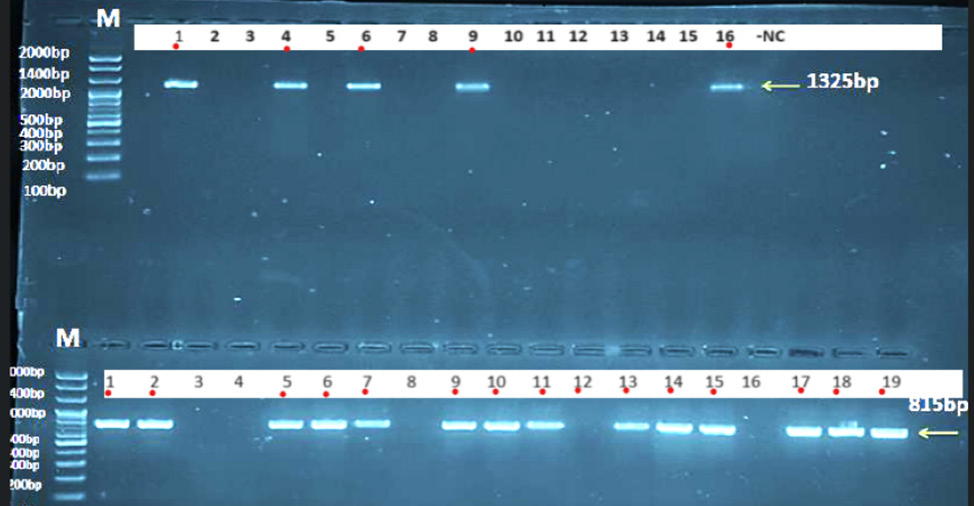
Agarose gel (1.5%) electrophoresis image that showed the First round (upper) PCR product analysis of conserved region in small subunit ribosomal RNA gene in *Cryptosporidium* species from Human stool samples. Where M: marker (2,000–100 bp). First round (upper), lanes (1–20), only 2, 5, 7, 10, 17 showed the first nPCR amplification that was positive for *Cryptosporidium* species samples at (1,325 bp) nPCR product. Second round (Lower) samples from 1–20 except 3, 4, 8, 12, 16 showed second nested PCR amplification that was positive for *Cryptosporidium* species samples at (815 bp) Nested PCR product.

Results of the two DNA fragments of ~1,325 bp and ~ 815 bp (Fig. 2). All homology sequence data identity between local *Cryptosporidium* spp. IQF-handlar No.1 to No.5 isolates submitted into NCBI GenBank and identified by accession numbers PP789667, PP789668, PP789669, PP789670, and PP789671 and GenBank-related *C*. *parvum* of Iran isolate showed sequence homology ranged from 99.45%–99.84%. The homology sequence identity between local *Cryptosporidium* spp. IQF-human numbers. Five isolates and NCBI-GenBank-related *C*.* parvum* China isolates conducted genetic homology sequence with identity varying from 99.45%–99.85%.

### Phylogenetic tree

**Figure 3.**
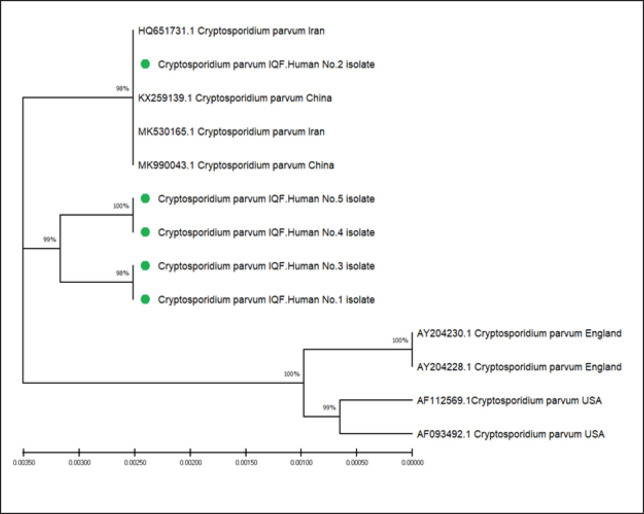
The phylogenetic tree constructed using the neighbor-joining method. Five *Cryptosporidium parvum* isolates (the sample from this study is marked with a green triangle) grouped in subclades with isolates from Iran and India. The tree is drawn to scale, with branch lengths measured in the number of substitutions per site (next to the branches).

Sequence alignment of all the obtained small subunit ribosomal RNA gene fragments in local *Cryptosporidium* species from human and NCBI-GenBank correspondent isolates. All multiple alignment analyses were performed through the online Clustal W Alignment profile. The phylogenetic tree construction was based on the partial sequence in local *C.* *parvum* human isolates used for geographical distribution and evolutionary comparison, as seen in Figure 3. The likelihood tree generated from the final alignment shows three major clades. The first one includes local and regional isolates from China and Iran with a high similarity. The second holds two clusters of five *C. parvum* from the current study, while the last clade, constricted species from England and the USA, has distinct branches as in Tables 4 and 5. The current molecular results presented parasite species similar to those in previous studies. For example, a study in Bangladesh reported *C*.* parvum* in poultry, with a very low percentage, found in 1.0% (2/197), and suggested that it can be a source of human infection [28]. A survey study in a hospital in Wasit Province in Iraq found a high prevalence of *C*.* parvum* in 60% (48/80) of the admitted patients [29].

**Table 2.** Prevalence rates of *Cryptosporidium* spp. in handlers related to a group of age.

Age/years	No. of examined	No. of infected	(%)
<15	6	2	33.3
*15*–35	27	14	51.8
3*6*–55	18	8	44.4
>55	9	3	33.3
Total	60	27	45
*p*-value 0.0001*			

* Significant differences.

**Table 3.** Infection rates of *Cryptosporidium* in fish handlers according to months.

Months	No. of the examined handler	No. of infected handlers	(%)
November	10	4	40.0
December	9	3	30.0
January	11	4	36.3
February	8	4	50.0
March	12	6	50.0
April	10	6	60.0
Total	60	27	45
*p*-value 0.0001*			

* Significant differences.

**Table 4.** The *Cryptosporidium parvum* human isolates and NCBI-BLAST closed genetic corresponding to isolates from China.

IQF-human *Cryptosporidium* sp. isolates	Accession numbers	Homology sequence identity (%)		
		Identical *Cryptosporidium* sp.	Accession numbers	Identity (%)
No.1	PP789667	*C*.* parvum* China	MK990043	99.55
No.2	PP789668	*C*.* parvum* China	KX259139	99.85
No.3	PP789669	*C*.* parvum* China	MK990043	99.55
No.4	PP789670	*C*.* parvum* China	MK990043	99.65
No.5	PP789671	*C*.* parvum* China	MK990043	99.85

**Table 5.** The *Cryptosporidium parvum* human isolates and NCBI-BLAST closed genetic corresponding isolates from Iran.

IQF-Human *Cryptosporidium* sp. isolates	Accession numbers	Homology sequence identity (%)		
		Identical *Cryptosporidium* sp.	Accession numbers	Identity (%)
No.1	PP789667	*C*.* parvum* Iran isolate	MK530165.1	99.45
No.2	PP789668	*C*.* parvum* Iran isolate	HQ651731.1	99.84
No.3	PP789669	*C*.* parvum* Iran isolate	MK530165.1	99.45
No.4	PP789670	*C*.* parvum* Iran isolate	MK530165.1	99.60
No.5	PP789671	*C*.* parvum* Iran isolate	MK530165.1	99.84

## Conclusion

The study enhanced our understanding of the prevalence and genetics of *Cryptosporidium *infection in fish handlers in Baghdad. However, much information remains that still needs to be clarified. The results emphasize some molecular and traditional investigations underlying the local and regional *Cryptosporidium* isolates. The sample population shows an infection in the parasite *Cryptosporidium* fish handler, including the fishermen, in November, December, January, February, March, and April. However, DNA fragment Sanger sequencing may reveal alien data about parasite isolates; the next-generation sequencing will considerably improve the current knowledge of the taxonomy, transmission, and geographical distribution of *Cryptosporidium* spp. by monitoring the emergence of different subtypes.

## List of abbreviations

NCBI, National Center for Biotechnology Information; PCR, polymerase chain reaction; SSU, small subunit.
